# Cell wall degradation is required for normal starch mobilisation in barley endosperm

**DOI:** 10.1038/srep33215

**Published:** 2016-09-13

**Authors:** Vasilios M. E. Andriotis, Martin Rejzek, Elaine Barclay, Michael D. Rugen, Robert A. Field, Alison M. Smith

**Affiliations:** 1 John Innes Centre, Norwich Research Park, Norwich NR4 7UH, United Kingdom

## Abstract

Starch degradation in barley endosperm provides carbon for early seedling growth, but the control of this process is poorly understood. We investigated whether endosperm cell wall degradation is an important determinant of the rate of starch degradation. We identified iminosugar inhibitors of enzymes that degrade the cell wall component arabinoxylan. The iminosugar 1,4-dideoxy-1, 4-imino-l-arabinitol (LAB) inhibits arabinoxylan arabinofuranohydrolase (AXAH) but does not inhibit the main starch-degrading enzymes α- and β-amylase and limit dextrinase. AXAH activity in the endosperm appears soon after the onset of germination and resides in dimers putatively containing two isoforms, AXAH1 and AXAH2. Upon grain imbibition, mobilisation of arabinoxylan and starch spreads across the endosperm from the aleurone towards the crease. The front of arabinoxylan degradation precedes that of starch degradation. Incubation of grains with LAB decreases the rate of loss of both arabinoxylan and starch, and retards the spread of both degradation processes across the endosperm. We propose that starch degradation in the endosperm is dependent on cell wall degradation, which permeabilises the walls and thus permits rapid diffusion of amylolytic enzymes. AXAH may be of particular importance in this respect. These results provide new insights into the mobilization of endosperm reserves to support early seedling growth.

Starch mobilisation in the endosperm of barley seedlings provides the substrate for early growth following grain germination. The process is understood in detail in terms of control of expression of genes encoding key enzymes, and the structure-function relationships of these enzymes^1^,^2^,^3^,^4^,^5^,^6^. However, the factors that control the rate of starch mobilisation during early seedling growth are poorly understood. Although it is generally agreed that α-amylase is responsible for the initial attack on starch granules, the extent to which the rate of synthesis of α-amylase in the scutellum and aleurone layer determines the rate of starch degradation during germination is not known. Some studies show a correlation between α-amylase activity and starch conversion to sugars during germination^7^, while others suggest that the rate of starch hydrolysis is not related to the absolute activity of amylases^8^.

It has long been speculated that the rate of starch mobilisation depends not on the rate of synthesis of α-amylase, but on the accessibility of endosperm starch granules to this enzyme following its release into the endosperm^8^,^9^,^10^,^11^,^12^. Endosperm cell walls are a potential barrier to the diffusion of hydrolytic enzymes from the aleurone and scutellum into the endosperm. During germination, cell-wall degrading enzymes and α-amylases diffuse from the proximal (scutellum) end of the endosperm towards the distal end, and from the aleurone towards the crease. A similar pattern and timing of spread is seen for the hydrolysis of cell wall β-glucans and starch^9^,^13^. The permeability of the endosperm to macromolecules is closely linked to cell wall hydrolysis^14^, and some approaches suggest that starch mobilisation occurs immediately behind the spreading front of cell wall degradation^9^. These observations have led some authors to assume that the rate of cell wall degradation determines the rate of diffusion of α-amylase through the endosperm, and hence the rate of starch mobilisation^15^,^16^,^17^. However, definitive information about the relationship between cell wall degradation and starch mobilisation is lacking.

In barley grains, about 70% of the mass of endosperm cell walls is (1,3;1,4)-β-d-glucans (referred to as β-glucan), and 20% of the mass is arabinoxylan (referred to as AX) consisting of β-1, 4-xylan chains with α-l-arabinofuranose substitutions at O-3 and/or O-2 positions of the xylanopyranoside (d-Xyl*p*). Lesser amounts of cellulose, glucomannan, xyloglucan, pectic polysaccharides, callose and arabinogalactan proteins are also present^10^,^11^,^18^. To discover whether loss of cell wall integrity is important for starch degradation, we looked for chemical inhibitors that would retard degradation of endosperm cell wall components without directly affecting enzymes of starch degradation during grain germination and early seedling growth. We demonstrate that incubation of barley grains with an iminosugar that inhibits a specific AX-degrading enzyme reduced both cell wall degradation and starch mobilisation. These results provide new and detailed insights into the way in which reserves in the barley endosperm are mobilised for seedling growth.

## Results

### Identification of iminosugar inhibitors of arabinoxylan-degrading enzymes

In order to test the effect of endosperm cell wall integrity on starch loss during barley grain germination and early seedling growth, we looked for inhibitors of enzymes that degrade components of the wall. We focussed on the degradation of the AX component, since this process requires several distinct classes of enzymes likely to be targets for different small molecule inhibitors. Four classes of enzymes may be involved in AX degradation in the endosperm of germinating barley grain. Endo-1, 4-β-d-xylan xylanohydrolase XYN-1 (referred to as endo-xylanase; EC 3.2.1.8; CAZy family GH10^19^) cleaves the xylan backbone of AX. Arabinoxylan arabinofuranohydrolase (AXAH) releases l-Ara*f* from the xylan backbone, and can also hydrolyse linkages between two arabinofuranose residues^20^,^21^. Arabinofuranosidase (ARA) and β-d-xylosidase are exo-acting enzymes that release terminal residues from oligosaccharides but attack AX only very slowly. Xylosidase is essentially specific for xylose residues; arabinofuranosidase will release both arabinose and xylose residues^22^,^23^,^24^,^25^.

We determined the profile of AX-degrading enzymes in the endosperm during germination and early seedling growth, using 4-nitrophenyl derivatives of substrates of these enzymes to measure activities in endosperm extracts. Activity of AXAH and/or arabinofuranosidase (not distinguishable in our assays; assayed with 4-nitrophenyl l-arabinofuranoside, pNPA) and β-d-xylosidase (assayed with 4-nitrophenyl β-d-xylanopyranoside, pNPX) increased sharply from about three days post-imbibition (dpi) but endo-xylanase activity (assayed with 4-nitrophenyl xylotrioside, OpNPX3) did not start to increase until five dpi (Supplementary Fig. S1a–c). The later appearance of endo-xylanase than AXAH is consistent with their modes of synthesis: while AXAH is produced in the endoplasmic reticulum of aleurone cells and secreted into the endosperm^21^, endo-xylanase is a cytosolic enzyme that is released into the endosperm only upon the programmed cell death of aleurone cells^19^,^24^.

**Figure 1:**
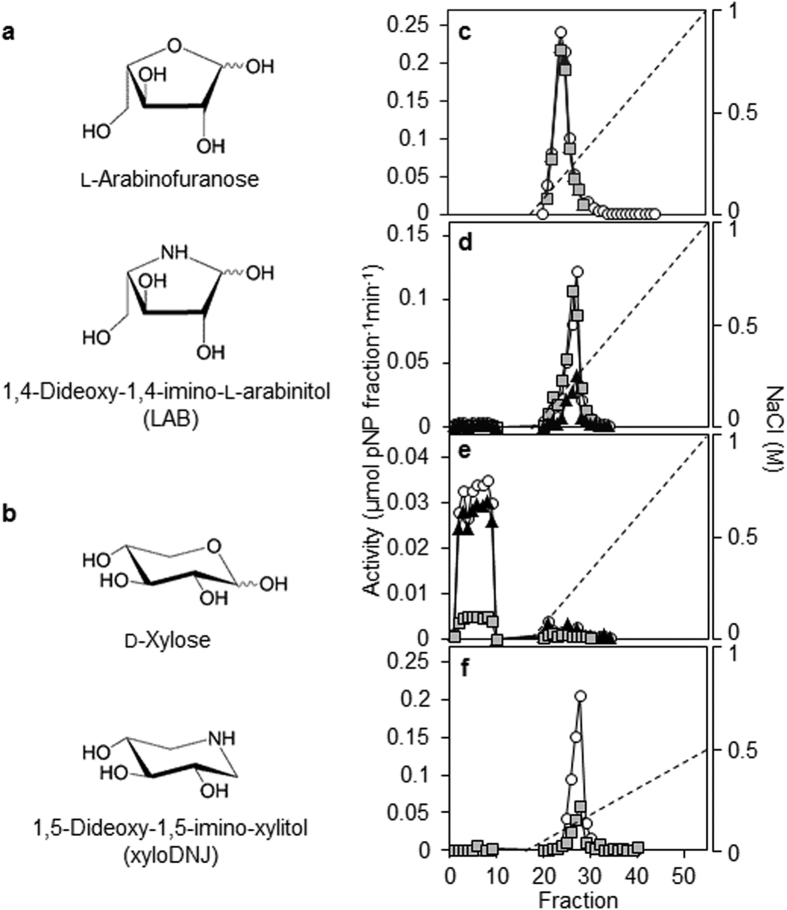
The iminosugar 1, 4-dideoxy-1, 4-imino-l-arabinitol (LAB) is an inhibitor of barley endosperm arabinoxylan arabinofuranohydrolase (AXAH) activity. (**a**) Structure of l-arabinofuranose (top) and LAB (bottom). (**b**) Structure of d-xylose (top) and of the iminosugar 1, 5-dideoxy-1, 5-imino-xylitol (xyloDNJ; bottom). (**c–f**) Chromatographic separation of barley endosperm AX degrading enzymes by FPLC. Soluble proteins from endosperm of seedlings at ten dpi were applied to a MonoQ anion exchange column (**c–e**). After washing, the column was eluted with a gradient of increasing NaCl concentration (dashed line). Proteins not binding on this column were applied to a MonoS cation exchange column (**f**) and eluted with a NaCl gradient (dashed line). Enzyme activities were assayed either in the absence (white circles) or in the presence of 500 μM LAB (black triangles) or xyloDNJ (grey squares). (**c**) Elution profile of endo-xylanase activity (assayed with 4-nitrophenyl xylotrioside). A single peak of endo-xylanase activity eluted from the MonoQ anion exchange column at about 200 mM NaCl. (**d**) Elution profile of AXAH activity (assayed with 4-nitrophenyl α-l-arabinofuranoside). AXAH eluted from the MonoQ anion exchange column at about 300 mM NaCl and was strongly inhibited by LAB. (**e**) Elution profile of xylosidase activity (assayed with 4-nitrophenyl xylanopyranoside). Xylosidase activity did not bind to the MonoQ anion exchange column. (**f**) Elution profile of xylosidase during MonoS cation exchange chromatography. Xylosidase activity that did not bind to a MonoQ anion exchange column (**e**) was applied to a MonoS cation exchange column and eluted as a single peak at about 150 mM NaCl.

To attempt to reduce the rate of AX degradation in the endosperm, we investigated whether iminosugars – established inhibitors of glycosidases – could inhibit specific classes of these enzymes^26^. The iminosugar 1,4-dideoxy-1, 4-imino-l-arabinitol (LAB) structurally resembles l-Ara*f* substituents of the xylan backbone of AX (Fig. 1a) and is an established inhibitor of a fungal arabinofuranosidase^27^. The iminosugar 1,5-dideoxy-1, 5-imino-xylitol (xyloDNJ) that structurally resembles d-Xyl*p* (Fig. 1b) is an inhibitor of bacterial β-d-xylosidase^28^. Neither LAB nor xyloDNJ is reported to affect plant glycohydrolases. Iminosugar inhibitors of endo-xylanases have been reported^29^ but were not available for this study, and no specific inhibitors of AXAH have been reported. We focussed our efforts on the monosaccharide mimetics LAB and xyloDNJ because other potential inhibitors are of larger molecular size and are likely to present uptake problems. Note that while the pseudotetrasaccharide acarbose is a potent inhibitor of barley α-amylase *in vitro*^30^, it does not affect barley grain germination even at millimolar concentrations (data not shown), likely because its size and/or polarity restrict uptake into the grain.

The inhibitory effects of LAB and xyloDNJ were tested on enzymes partially purified from extracts of endosperm at ten dpi. A concentration of 500 μM was used, since this concentration of the iminosugar deoxynojirimycin (DNJ) is sufficient to inhibit starch degradation when applied to intact, germinating barley grains^31^. Endo-xylanase activity bound to an anion exchange column (MonoQ) and was eluted in a salt gradient as a single peak (Fig. 1c). This activity was not inhibited by LAB or xyloDNJ. Most of the AXAH/arabinofuranosidase activity (assayed with pNPA) bound to the MonoQ column, and eluted in a salt gradient. LAB inhibited this activity by almost 70% whereas xyloDNJ had no effect (Fig. 1d). This activity is likely to be due to AXAH rather than arabinofuranosidase. First, the peak contained very little xylosidase activity: when assayed with the xylosidase substrate pNPX, activity was only ~2% of that with pNPA (compare Fig. 1c,e). AXAH is unable to hydrolyse pNPX^20^,^32^ whereas the only arabinofuranosidase characterised from germinating barley grain, ARA-I, is a bifunctional enzyme able to hydrolyse pNPX at about 20% of the rate of pNPA hydrolysis^23^. Second, there is little evidence for the presence of ARA-I in the endosperm of germinating grains. ARA-I transcript was absent from aleurone and scutellum^23^, and ARA-I protein was not detected in a proteomic survey of secreted and internal proteins from aleurone layers treated with gibberellin whereas AXAH proteins were present in both fractions^33^.

Most of the β-d-xylosidase activity in endosperm extracts did not bind to the MonoQ column, but bound to a cation exchange column (MonoS) and eluted as a single peak (Fig. 1e,f). This activity was 85% inhibited by xyloDNJ, but was not inhibited by LAB. We also tested LAB against partially purified activities of other glycosidases that might be involved in endosperm mobilisation, using nitrophenyl glycosides as substrates. LAB did not inhibit β-d-glucosidase, α-mannosidase, or β-galactosidase activities (Supplementary Fig. S2).

### LAB inhibits seedling growth and starch mobilisation

**Figure 2:**
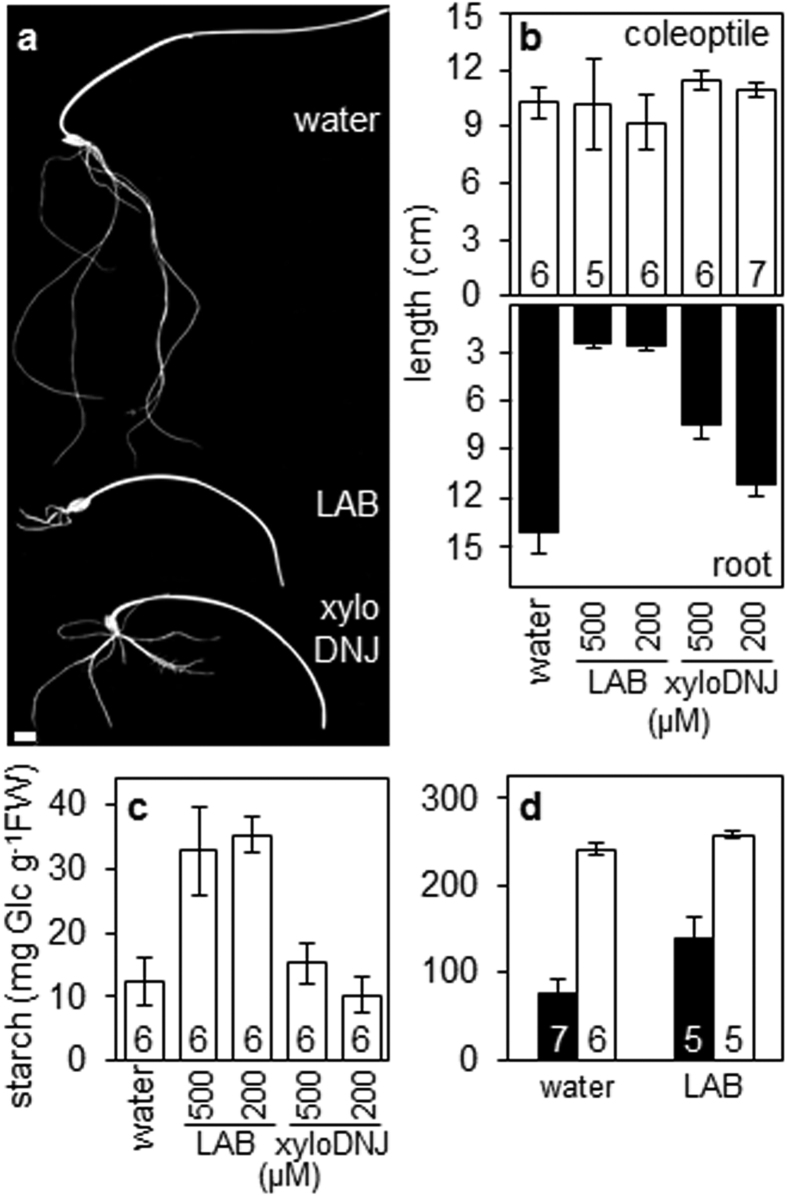
Effects of LAB and xyloDNJ on seedling growth and endosperm starch content. (**a**) Appearance of seedlings of cv NFC Tipple at ten dpi, grown in water alone or in the presence of 500 μM LAB or xyloDNJ. Bar = 1 cm. (**b**) Effect of inhibitors on coleoptiles (top panel) and roots (bottom panel) of seedlings at ten dpi grown in water alone or in the presence of 200 or 500 μM of each inhibitor. Values are means ± SE of measurements made on the numbers of seedlings indicated in the top part of the graph. (**c**) Endosperm starch content of barley seedlings at ten dpi grown in water alone or in the presence of the indicated levels (μM) of LAB or xyloDNJ. Values are means ± SE of measurements made on the number of seedlings indicated. (**d**) Effect of LAB on starch degradation in endosperm of embryo-less half-grains. Embryo-less half-grains were incubated with water or in the presence of 500 μM LAB, either supplemented with (closed bars) or lacking (open bars) gibberellin. Starch content was measured at eight dpi. Values are means ± SE (bars) of measurements on the number of half-grains indicated.

Barley grains were allowed to germinate from the point of imbibition in the presence or absence of LAB or xyloDNJ. Root growth of seedlings was severely inhibited by LAB at 200 μM (more than 80% inhibition relative to control seedlings growing in water only; Fig. 2a,b; Supplementary Fig. S3). Roots ceased to grow at about four dpi and became brown. LAB did not affect coleoptile growth. XyloDNJ also reduced root growth, but to a lesser extent than LAB (Fig. 2a,b). At 500 μM, xyloDNJ reduced root growth by less than 50% relative to control seedlings growing in water only (Fig. 2b; Supplementary Fig. S3).

LAB inhibited starch mobilisation in the endosperm. At ten dpi, control seedlings contained 12 mg starch g^−1^ fresh weight whereas LAB-treated seedlings contained almost three times this amount. XyloDNJ had little effect on starch mobilisation: at ten dpi the starch contents of xyloDNJ-treated and control seedlings were similar (Fig. 2c).

The inhibition of both root growth and starch mobilisation by LAB raises the possibility that these effects are causally related. We first considered the possibility that the effects of LAB on endosperm starch mobilisation are secondary consequences of its inhibition of root growth. For example, starch degradation might be inhibited by a build-up of degradation products in the absence of their utilisation for root growth. To investigate this possibility, we examined the effects of LAB on starch mobilisation in half-grains, lacking the embryo and hence the capacity for rapid metabolism of the products of starch degradation. Starch mobilisation in half-grains incubated in water was greatly accelerated by the addition of gibberellin (GA) (Fig. 2d). GA also accelerated starch mobilisation in half-grains incubated in the presence of LAB, but to a lesser extent. After eight days, GA-treated half-grains incubated with LAB had twice as much starch as GA-treated half-grains incubated in water only. This result shows that LAB directly affects processes in the endosperm: its action on starch mobilisation is not a secondary consequence of its inhibition of root growth.

### Effect of LAB on the activity of enzymes of starch degradation

**Figure 3:**
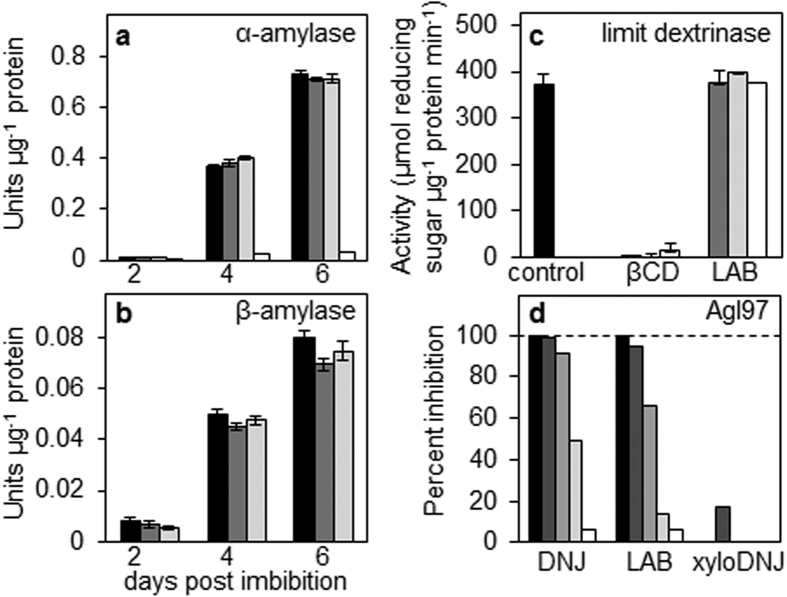
Effect of LAB on the activities of enzymes of starch degradation. (**a**) Effect of inhibitors on α-amylase activity. Extracts of endosperm from seedlings at the dpi indicated were assayed for α-amylase activity in the absence (black bars) or in the presence of 500 μM (dark grey bars) or 100 μM (light grey bars) LAB, or acarbose, a known inhibitor of the enzyme (500 μM; white bars). Values are means ± SE of measurements on three independent extracts. (**b**) Effect of inhibitors on β-amylase activity. Extracts as in (a) were assayed for β-amylase activity either in the absence (black bars) or in the presence of 500 μM (dark grey bars) or 100 μM (light grey bars) LAB. Values are means ± SE of measurements on three independent extracts. (**c**) Effect of inhibitors on limit dextrinase activity. Activity of purified recombinant limit dextrinase was assayed against pullulan in the absence of inhibitors (black bar) or in the presence of 500 μM (dark grey bars), 100 μM (light grey bars), or 50 μM (white bars) of LAB, or β-cyclodextrin (βCD), a known inhibitor of the enzyme. Values are means ± SE of three independent measurements. (**d**) Effect of inhibitors on maltase (Agl97) activity. Purified recombinant Agl97 was assayed with pNPG in the presence of 100 μM (black bars), 10 μM (dark grey bars), 1 μM (grey bars), 0.1 μM (light grey bars), or 0.01 μM (white bars) of LAB, xyloDNJ or deoxynojirimycin (DNJ), a known inhibitor of the enzyme^31^. Results are expressed as percent inhibition relative to control assays with no inhibitor. The enzyme preparation was as previously specified^31^.

We tested whether LAB affects starch mobilisation through an unanticipated direct effect on starch degrading enzymes. Activity of α-amylase in extracts from barley seedlings was unaffected by LAB (Fig. 3a) whereas acarbose – known to inhibit α-amylases^30^ – drastically reduced α-amylase activity. LAB also had little effect on the activity of β-amylase (Fig. 3b). LAB did not affect the activity of purified recombinant limit dextrinase (Fig. 3c) whereas the limit dextrinase inhibitor β-cyclodextrin^6^ almost completely inhibited limit dextrinase under similar conditions.

LAB inhibited the activity of purified recombinant maltase (Agl97), the enzyme responsible for the conversion of starch-derived maltose to glucose in the endosperm (Fig. 3d). However, inhibition of maltase is highly unlikely to account for the inhibitory effect of LAB on starch mobilisation in the endosperm. First, we showed previously that transgenic barley grains with strong reductions in Agl97 expression have normal rates of starch degradation during germination^31^. Second, purification of maltase activity from barley endosperm failed to identify additional maltases and provided good evidence that essentially all of the maltase activity during grain germination and early seedling growth is accounted for by Agl97^34^. Third, a previous study of proteins either present in or secreted from isolated barley aleurone cell layers treated with GA failed to identify any maltases other than Agl97^33^. We conclude that the effects of LAB on starch degradation in barley endosperm are indirect, rather than the result of direct inhibition of starch-degrading enzymes.

### LAB reduces the extent of arabinoxylan degradation in endosperm cell walls

**Figure 4:**
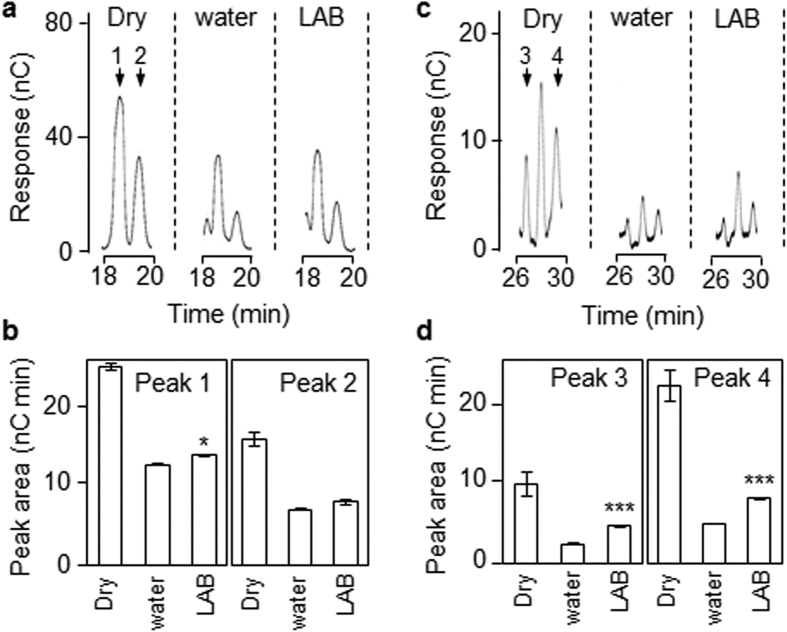
LAB reduces the rate of arabinoxylan degradation in endosperm cell walls. A cell wall fraction prepared from pooled endosperm from 30–40 grains was treated with commercial endo-xylanase and the released oligosaccharides were analysed by HPAEC. (**a**) Representative elution profiles of AX-diagnostic peaks 1 and 2 (arrows). The full elution profile is shown in Fig. S4. (**b**) Quantification of peak area of AX-diagnostic peaks 1 and 2 from the elution profiles in (**a**). Values are means ± SE from three technical replicates. Differences between inhibitor-treated and water incubated grains at six dpi are statistically significant as indicated (Student’s t-test: *P < 0.05). (**c**) As in (**a**) but for AX-diagnostic peaks 3 and 4. Elution profiles are from the same chromatograms as in (**a**). The full elution profile is shown in Fig. S4. (**d**) Quantification of peak area of AX-diagnostic peaks 3 and 4 from the elution profiles in (**c**). Values are means ± SE from three technical replicates. Differences between inhibitor-treated and water incubated grains at six dpi are statistically significant as indicated (Student’s t-test: ***P < 0.001).

We used two approaches to investigate whether AX degradation in the endosperm was reduced when grains germinated in the presence of LAB. First, endosperm cell wall preparations from dry grains were treated with bacterial endo-xylanase then fractionated by high-performance anion-exchange chromatography (HPAEC). To identify which peaks corresponded to AX oligosaccharides, some samples were treated with bacterial AXAH prior to fractionation. This treatment resulted in major alterations in the oligosaccharide profile detected by HPAEC, and in the accumulation of free arabinose and xylose (Supplementary Fig. S4a,b). Four peaks that were strongly reduced in size by AXAH treatment were used as diagnostics for AX composition/content in endosperm cell walls (Supplementary Fig. S4a). Fractionation of endo-xylanase-treated walls from grains at six dpi showed that all four peaks were much smaller than in dry grain, consistent with AX degradation in the endosperm during this period (Fig. 4). Three of the four peaks were larger in LAB-treated grains than in grains treated with water alone (peaks 1, 3 and 4), consistent with the idea that LAB retarded loss of AX from the endosperm by acting on AXAH (Fig. 4b,d).

**Figure 5:**
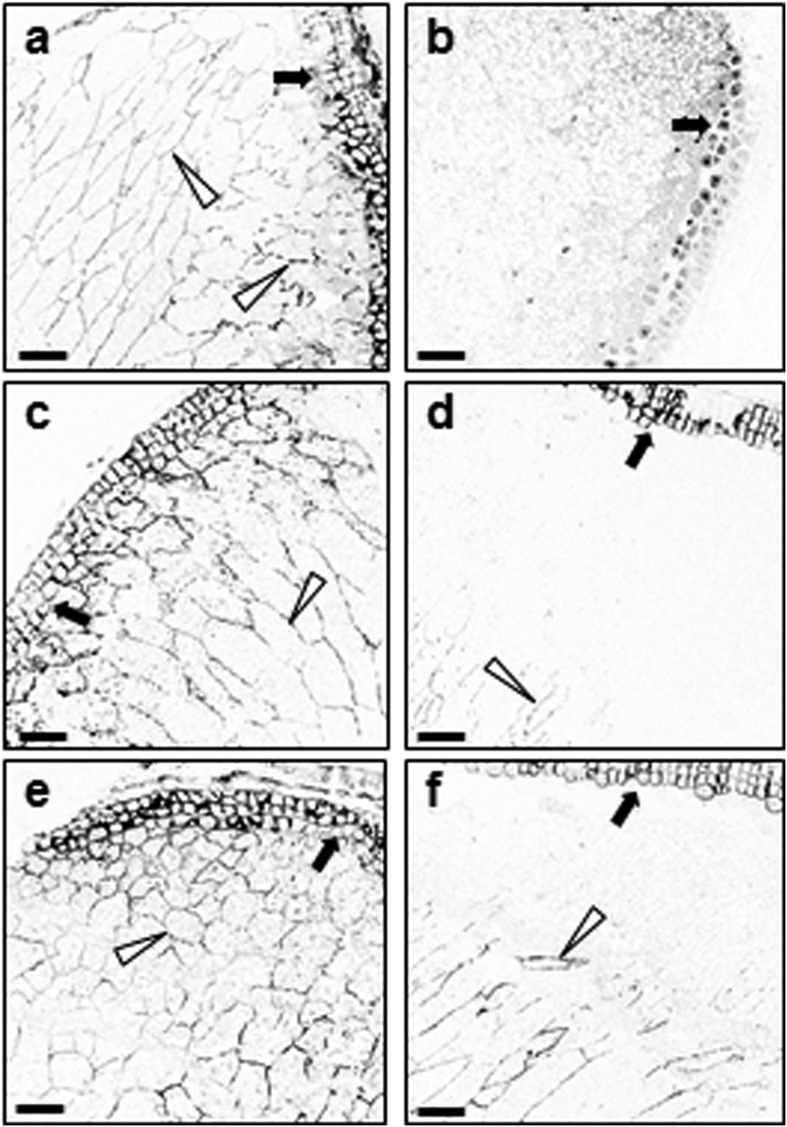
Treatment with LAB reduces the extent of arabinoxylan degradation in endosperm cell walls. Transverse sections of endosperm of dry grains (**a**,**b**), or grains imbibed and grown in water (**c**,**d**), or LAB (500 μM; **e**,**f**) were incubated with a monoclonal antibody against AX (LM11) then with an Alexa Fluor^®^ 633 conjugated secondary antibody, and subjected to confocal microscopy to visualise AX epitopes. All sections were prepared from approximately the same position of the grain. Open arrowheads point to fluorescence from AX epitopes in endosperm cell walls, closed arrows point to the aleurone layer. Original confocal images were edited such that colour was inverted in all panels at the same time, for optimal contrast between fluorescence signal and background. (**a**) Section through a dry grain, adjacent to the aleurone. The antibody decorates the cell walls of the endosperm and aleurone cells. (**b**) As in (**a**) but omitting the primary (LM11) antibody. No staining of cell walls is visible. (**c**) Section of barley grain grown in water, at two dpi. The distribution of fluorescence is the same as in dry grain (**a**). (**d**) Section of barley grain grown in water, at four dpi. Fluorescence is lower in endosperm cell walls adjacent to the aleurone than at two dpi. (**e**) Section of barley grain grown in LAB, at two dpi. The distribution of fluorescence is similar to that in dry grain (**a**). (**f**) Section of barley grain grown in water, at four dpi. Fluorescence is greater in the region adjacent to the aleurone layer than in grains at the same stage grown in water (**d**). Scale bars are 100 μm throughout.

**Figure 6:**
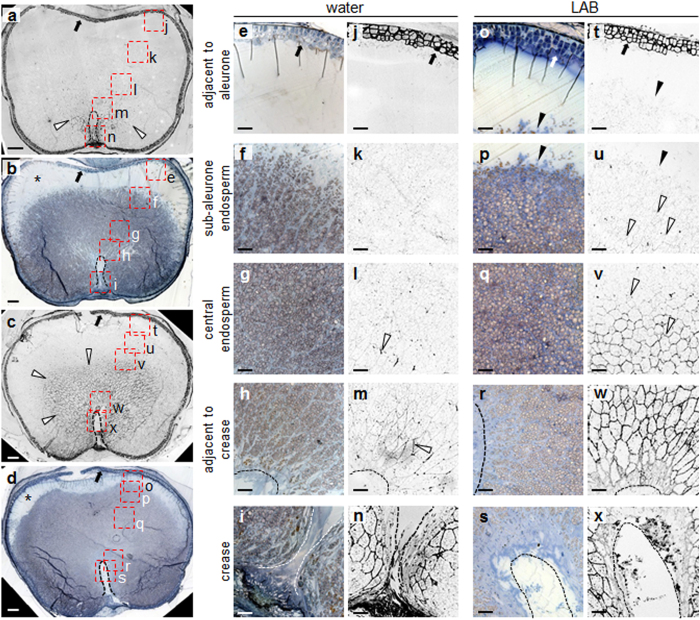
Treatment with LAB reduces the extent of both arabinoxylan and starch degradation in the endosperm. Transverse sections of endosperm of grains incubated for six dpi in water (**a**,**b**,**e–n**), or LAB (**c**,**d**,**o–x**) were either incubated with the LM11 antibody and processed as in Fig. 5, to visualise AX epitopes (**a**,**c**,**j–n**,**t–x**), or stained with toluidine blue and iodine solution (**b**,**d**,**e–i**,**o–s**) to visualise cellular structures and starch deposits. All sections were prepared from approximately the same position of the grain. Open arrowheads: fluorescence from AX epitopes in endosperm cell walls; closed arrowheads: interface between the sub-aleurone zone and endosperm tissue in which starch degradation has been initiated; closed arrows: aleurone layer; dashed lines (**a–d**,**h**,**i**,**m**,**n**,**r**,**s**,**w**,**x**) edge of the crease; asterisks (**b**,**d**): sub-aleurone zone. Results were reproducible in two separate incubations. In (**a–d**) letters next to the red boxes correspond to panels (**e–x**) as indicated. (**a**) Section through a grain grown in water, incubated with the LM11 antibody and viewed under a confocal microscope. The antibody decorates the cell walls of the endosperm cells around the crease of the grain, and the aleurone. (**b**) Section consecutive to that shown in (**a**), stained with toluidine blue and iodine solution. (**c**) As in (**a**) but for a grain grown in LAB. The LM11 antibody decorates cell walls in a much larger area of the endosperm surrounding the crease, compared to (**a**). (**d**) As in (**b**) but for a grain grown in LAB. Loss of starch from the sub-aleurone zone has not progressed to the same extent as in (**b**). (**e–n**) Close-up views of a section through a grain grown in water. The sections correspond to the five boxed areas of the grain shown in (**a**),(**b**). (**e–i**) were stained with toluidine blue and iodine to reveal starch; (**j–n**) were stained with LM11 antibody to reveal AX epitopes. (**o–x**) As in (**e–n**) but for a grain grown with LAB. (**o–s**) were stained with toluidine blue and iodine to reveal starch; (**t–x**) were stained with LM11 antibody to reveal AX epitopes. Scale bars are 100 μm (**a–d**) and 50 μm (**e–x**).

Second, we used immunofluorescence microscopy to determine the distribution of AX in endosperm cell walls during seedling growth up to six dpi. The monoclonal antibody LM11 recognises unsubstituted and relatively low-substituted xylans and binds strongly to wheat endosperm AX^35^. It has been used recently to follow AX accumulation in the endosperm cell walls of developing wheat grains^36^. In sections from dry barley grains, the antibody decorated the cell walls of the aleurone and the endosperm (Fig. 5a). No fluorescence signal was detected when the primary antibody was omitted (Fig. 5b). The distribution of fluorescence was unaltered at two dpi in grains incubated in water. After four days, fluorescence was greatly reduced or absent from endosperm cells adjacent to the aleurone, suggesting that AX degradation commences in this region of the endosperm (Fig. 5c,d). At six days, fluorescence was detectable only in the walls of cells surrounding the crease of the grain (Fig. 6a).

Incubation of grains with LAB substantially retarded the loss of fluorescence from endosperm cell walls. The presence of LAB had little effect on the distribution of fluorescence up to two dpi (Fig. 5e), but at four dpi fluorescence was greater in the region adjacent to the aleurone layer in LAB-treated grains than in those in water only (control grains; Fig. 5f). After six days the fluorescent region of the endosperm adjacent to the crease was substantially greater in LAB-treated grains than in control grains (Fig. 6c, Supplementary Fig. S5). Thus LAB retards the rate at which a wave of degradation of AX spreads across the endosperm from the aleurone towards the crease during germination.

### Cell wall arabinoxylan degradation precedes starch mobilisation

To visualise the pattern of starch mobilisation in the endosperm, sections from grains at six dpi were stained with iodine solution. At this time point no cellular structures were visible in the endosperm adjacent to the aleurone in grains incubated in water only, indicating extensive mobilisation of all cellular material (Fig. 6b,e). Starch granules were present at the interface between the sub-aleurone zone and the central endosperm (Fig. 6f). These granules were small and fragmented, indicating that they were partially digested. Immunofluorescence microscopy failed to detect AX in the sub-aleurone and interface zones (Fig. 6j,k). Starch was also present in sections through the central endosperm toward the crease of the grain (Fig. 6g–i). Fluorescence from AX epitopes was low throughout this zone, increasing in intensity immediately around the crease of the grain (Fig. 6l–n). Incubation of grains with LAB reduced the width of the sub-aleurone zone from which cellular structures had been lost (compare Fig. 6b,d,e,o). A much greater area of the endosperm retained starch than was the case for grains incubated in water alone. At the interface between the sub-aleuronal and the central endosperm starch granules were small and fragmented as in grains incubated in water only (Fig. 6p). Fluorescence from AX epitopes was extremely weak or absent at the interface and immediately below it, but in the central endosperm generally it was much higher than for grains incubated in water only (Fig. 6v–x). These results show that AX degradation precedes major loss of starch, and that retardation by LAB of the spread of AX degradation from the aleurone to the crease is accompanied by a similar pattern of retardation of starch degradation.

### Endosperm AXAH activity resides in oligomeric protein complexes

**Figure 7:**
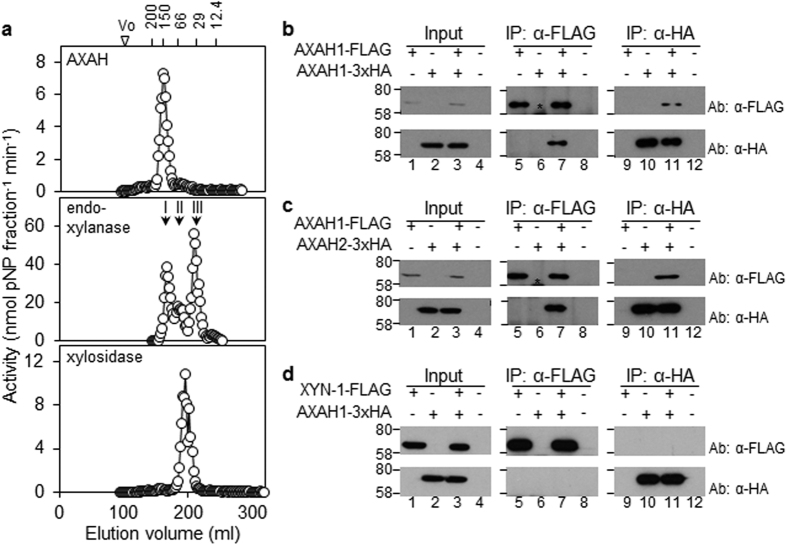
Size fractionation of AXAH, endo-xylanase and β-d-xylosidase from endosperm extracts, and detection of oligomeric protein complexes. (**a**) Size fractionation of AX-degrading enzymes from endosperm extracts of barley seedlings at ten dpi were applied to a Sephacryl S200 column by FPLC. Fractions were assayed for AXAH (top panel), endo-xylanase (middle panel) and β-d-xylosidase (bottom panel) activity. Molecular masses of eluted activities were estimated by comparison to the elution profile of proteins of known size (numbers at the top, in kDa). (**b**) AXAH1 can homodimerise. AXAH1-FLAG and/or AXAH1-3xHA proteins were transiently expressed in *N. benthamiana* leaves. +Above a lane indicates that the protein indicated at the left was expressed in the leaf from which extracts were made. Input samples are extracts prior to immunoprecipitation; IP: α-FLAG and IP: α-HA are proteins precipitated with the FLAG and HA antisera respectively. Ab: α-FLAG and Ab: α-HA indicate the antiserum used for immunodetection of proteins following SDS-PAGE. Numbers on the left are positions of molecular mass markers (in kDa). Numbers below each panel indicate lane numbers. Samples were subjected to electrophoresis on the same gel and to immunoblot analysis at the same time. Asterisk: position of the IgG heavy chain occasionally detected by the antisera. (**c**) AXAH1 interacts with AXAH2. AXAH1-FLAG and/or AXAH2-3xHA were transiently expressed in *N. benthamiana* leaves. Annotation is as for (**b**). (**d**) AXAH1 does not interact with XYN-1. AXAH1-3xHA and/or XYN-1-FLAG were transiently expressed in *N. benthamiana* leaves. Annotation is as for (**b**).

Given the apparent importance of AX degradation for starch mobilisation in the endosperm, we investigated further the nature of the enzymes responsible for this process. We used size exclusion chromatography to fractionate extracts of endosperm during seedling establishment (4–10 dpi). A single AXAH activity peak was detected in all samples (Fig. 7a; Supplementary Fig. S6a). Its estimated size was about 140 kDa, approximately twice the predicted size for barley AXAH^21^. Endo-xylanase activity in extracts from seedlings at four and six dpi eluted as a single peak with an estimated size of about 125 kDa (peak I; Supplementary Fig. S6b). Two additional peaks of endo-xylanase activity were detected in samples from older seedlings, with estimated sizes of 60–68 kDa (peak II) and about 30 kDa (peak III; Fig. 7a; Supplementary Fig. S6b). None of these activity peaks corresponded to xylosidase: this activity eluted as a single peak with an estimated size of about 42 kDa (Fig. 7a).

These results indicated that AXAH and endo-xylanase activities in the endosperm may reside in multimeric complexes. Examination of publicly-available data on transcript levels of AXAH and endo-xylanase in barley indicates that the major isoforms present in the endosperm during grain germination and seedling growth may be XYN-I, and AXAH1 and AXAH2 respectively. Five genes in barley encode AXAH^21^. Of these *AXAH1* and *AXAH2* are much more highly expressed in the embryos of germinating seeds than *AXAH3* or *AXAH4*, and *AXAH5* transcripts are almost undetectable in this organ^21^,^37^. Two genes in barley encode endo-xylanases. *XYN-1* is strongly expressed in the aleurone, whereas *XYN-2* (also referred to as X-II^38^) is expressed in the shoot and root of seedlings^19^,^22^,^38^.

To investigate the occurrence of homo- or hetero-multimeric complexes containing AXAH and/or endo-xylanase, AXAH1, AXAH2 and XYN-1 were transiently expressed in *Nicotiana benthamiana* leaves, as chimeric proteins with either FLAG, hemagglutinin (3xHA) or c-Myc (3xcMyc) epitope tags at the C-terminus. Chimeric proteins accumulated only in infiltrated areas of the leaf. They were active enzymes, and they could be specifically and quantitatively precipitated from leaf extracts with affinity matrices for the corresponding epitope tag (Supplementary Fig. S7).

To investigate multimer formation by AXAH1, we co-expressed AXAH1-FLAG and AXAH1-3xHA in *N. benthamiana* leaves, and treated extracts with an anti-FLAG (α-FLAG) affinity matrix. Immunoblot analysis of immunoprecipitated proteins with α-FLAG and anti-HA (α-HA) antibodies showed that both AXAH1-FLAG and AXAH1-3xHA were present (Fig. 7b, lane 7). This interaction was specific because no band corresponding to AXAH1-3xHA was detected after α-FLAG immunoprecipitation of extracts from leaves expressing AXAH1-3xHA alone, or from un-infiltrated areas of the leaf (Fig. 7b, lanes 6, 8). Reciprocal experiments using an α-HA affinity matrix recovered both AXAH1-3xHA and AXAH1-FLAG only when both proteins were transiently co-expressed (Fig. 7b, lane 11). These results show that AXAH1 can form homo-multimers. Similar experiments with FLAG- and 3xHA-tagged versions of AXAH2, and with -FLAG and -3xcMyc versions of XYN-1, showed that both of these proteins can also form homomultimers (Supplementary Fig. S8a,b).

AXAH1 and AXAH2 share more than 80% sequence identity at the amino acid level. To test whether they interact *in vivo*, AXAH1-FLAG and AXAH2-3xHA were co-expressed in *N. benthamiana* leaves. Immunoprecipitation experiments with either α-FLAG or α-HA affinity matrices recovered both AXAH1-FLAG and AXAH2-3xHA (Fig. 7c, lanes 7, 11), suggesting that AXAH1 and AXAH2 can interact *in vivo*.

We found no evidence for interactions between AXAH and XYN-1 when expressed in *N. benthamiana* leaves. Co-expression of XYN-1-FLAG with AXAH1-3xHA or AXAH2-3xHA and immunoprecipitation with specific affinity matrices did not recover any AXAH-XYN1 protein complexes. Although the α-FLAG affinity matrix specifically bound pXYN1-FLAG from extracts of infiltrated leaves, no band corresponding to either AXAH1-3xHA or AXAH2-3xHA was detected (Fig. 7d, lane 7; Supplementary Fig. S8c). Similar results were obtained when expressed proteins were immunoprecipitated with an α-HA affinity matrix (Fig. 7d, lane 11; Supplementary Fig. S8c). Therefore, XYN-1 does not physically interact with AXAHs under these conditions.

## Discussion

In order to examine the importance of cell wall degradation for the mobilisation of starch in the endosperm of barley grains, we looked for small-molecule inhibitors active against important enzymes of cell wall degradation but not against enzymes directly involved in starch degradation. The iminosugar LAB met these criteria. It inhibited the AX-degrading enzyme AXAH but not the endo-xylanase and xylosidase also implicated in AX degradation. Importantly it did not inhibit key enzymes of starch degradation including α-amylase, the enzyme responsible for the initial attack on starch granules, and β-amylase and limit dextrinase, enzymes which further process the products of α-amylolysis. LAB inhibited the downstream enzyme maltase, but this inhibition is highly unlikely to be responsible for its effects on starch degradation. First, transgenic lines of barley carrying an RNA interference silencing cassette for *HvAgl97*, the sole gene encoding maltase, have greatly reduced maltase activity and greatly reduced ratios of glucose to maltose, but retain normal rates of starch mobilisation^31^,^34^. Second, inhibition of maltase in germinating barley grains by incubation with the maltose analogue 4-*O*-α-glucosyl-moranoline (G1M) also resulted in a substantial reduction in the ratio of glucose to maltose but had little or no effect on starch degradation^31^. The concentration of G1M used in the incubation was fifty-fold greater than that required to achieve 60% inhibition of purified maltase.

Despite its lack of direct effect on enzymes important for starch degradation, the application of LAB to germinating barley grains markedly reduced the extent of degradation of both AX and starch over the first ten dpi. Two independent lines of evidence support this conclusion. First, direct measurements of endosperm starch content and the amounts of some specific products of AXAH degradation of endosperm cell walls show that incubation of grains with LAB retarded the rate of loss of both starch and AX during the first six to ten dpi. Second, visualisation of the distributions of starch and AX in endosperm sections revealed that incubation of grains with LAB retarded the spread of a wave of degradation of both polymers that proceeded from the aleurone towards the crease in the first six dpi. These spatial analyses also revealed that major loss of starch from regions of the endosperm was preceded by substantial or complete loss of AX. Taken together, these results are consistent with the view that LAB retards loss of AX from the endosperm, and does so specifically via its inhibitory effect on AXAH, and that the progression of starch degradation across the endosperm follows the loss of AX.

The fact that LAB retards the loss of both AX and starch from the endosperm suggests strongly that permeabilisation of cell walls through degradation of cell wall polymers is a prerequisite for starch degradation. It seems highly likely that the diffusion of α-amylase from the aleurone into the endosperm is prevented or strongly retarded by intact cell walls. Previous authors have shown that the temporal and spatial patterns of cell wall and starch degradation are similar and proposed that cell wall degradation may be important for starch degradation^39^, but our data constitute the first experimental evidence for a causal relationship between these processes.

Our results do not rule out the possibility that LAB retards loss of cell wall integrity by inhibiting more than one key enzyme of cell wall degradation. We showed that it does not inhibit endo-xylanase, β-d-xylosidase, β-d-glucosidase, α-d-mannosidase and β-d-galactosidase activities, but we have not examined its effects on enzymes that degrade β-glucans. However the clear and strong effects of LAB on AXAH activity *in vitro* and on AX and starch loss *in vivo* are consistent with the idea that AX degradation per se has a major impact on cell wall permeability and integrity, and that AXAH is a key enzyme for endosperm mobilisation as a whole. It is widely acknowledged that the arabinose substituents hinder the hydrolysis of the xylan backbone of arabinoxylan during its degradation. Enzymes that remove these substituents act synergistically with endo-xylanase in the complete degradation of the polymer^32^,^40^, and may alter arabinoxylan properties and interactions with other cell wall components^11^. AXAH is thus likely to be a crucial first step in arabinoxylan degradation, because of both its early production relative to endo-xylanase and its facilitation of endo-xylanase action.

The known synergistic interactions during arabinoxylan degradation between endo-xylanases and enzymes that remove arabinose substituents of AX prompted us to investigate whether actions of AXAH and endo-xylanase may be coordinated through protein-protein interactions.

The two gene products likely to account for AXAH activity in the endosperm, AXAH1 and AXAH2, are 83% identical, but nonetheless have different affinities for AX and arabinan substrates^21^. We showed that AXAH exists as a dimer in endosperm extracts, and that the two isoforms can interact to form both homo- and hetero-dimers. Related family GH51 enzymes from microorganisms also exist as multimers (usually hexamers) in solution^25^. The prevalence of homo- versus hetero-dimer forms during endosperm cell wall mobilisation and differences in catalytic properties between the heterodimer and the homodimers remain to be investigated.

In line with previous reports^19^,^24^,^41^, we found that endo-xylanase activity in the endosperm is initially associated with a single protein of relatively high mass, then active proteins of smaller masses appear as mobilisation proceeds. We showed that the unprocessed form of endo-xylanase forms homodimers. However, the same technique failed to demonstrate any interaction between AXAH and endo-xylanase. Thus although their actions are likely to be synergistic, it appears that the two enzymes act independently at a physical level.

The consequences of AXAH action for the integrity, permeability and susceptibility to degradation of the endosperm cell wall as a whole are difficult to predict because of the paucity of information about wall structure. Based on the ability of xylanases and arabinofuranosidase to solubilise β-glucans from endosperm cell walls, Bamforth and colleagues proposed that the endosperm cell wall consists of β-glucan sandwiched between outer layers of arabinoxylan^42^,^43^,^44^. If this is correct, removal of arabinoxylan side chains might be expected to accelerate loss of cell wall integrity as a whole, by exposing the inner β-glucan to attack by endoglucanases. However recent evidence suggests that wall structure may not conform to the Bamforth model. β-Glucan is now known to be synthesised at the plasma membrane and extruded into the apoplast, whereas arabinoxylans are made in the endomembrane system and secreted into the apoplast^45^. Consistent with this location of synthesis, staining techniques indicate that β-glucan may be preferentially located in regions of the cell wall adjacent to the plasma membrane in barley endosperm cells^46^. β-Glucan is proposed to act as a thick hydrogel that coats the surfaces of, and/or acts as a filler between, other insoluble cell wall constituents including arabinoxylans^47^,^48^. It may have a primarily metabolic role in the endosperm, as a source of glucose for embryo growth following germination, whereas arabinoxylan may have an important structural function. It is not known which of these two major wall components is the more important in determining permeability to proteins such as α-amylase.

The extent of degradation of cell walls during the malting of barley (the level of modification) is very important for malt quality. High levels of degradation are generally desirable because degradation products are available for yeast fermentation, and because partially degraded cell walls lead to high viscosity and impeded filtration at later stages of beer production^49^. Our results raise the possibility that selection of barley varieties with high levels and/or early production of AXAH might maximise the extent of cell wall degradation during malting and thus improve malt quality and the subsequent filterability of wort. However, it is also possible that elevated arabinoxylan-degrading activity during malting might accelerate starch degradation, leading to a loss of fermentable material. Further work will be required to establish whether the rate of arabinoxylan degradation is limiting for starch degradation in the malting process, and hence whether elevation of AXAH will lead to overall improvement of malt quality.

## Materials and Methods

### Plant material and inhibitors

Barley was the UK elite variety NFC Tipple. XyloDNJ was from GlycoSyn (www.glycosyn.com) and acarbose from Toronto Research Chemicals Inc. (http://www.trc-canada.com/). DNJ was a kind gift from Dextra Laboratories Ltd or purchased from Callaghan Innovation Research Ltd (http://www.irl.cri.nz/), LAB (1,4-dideoxy-1, 4-imino-l-arabinitol) was chemically synthesised from d-xylose^50^. Chromatographic columns were from GE Healthcare (http://www3.gehealthcare.co.uk/). Grains were surface-sterilised^31^ then germinated on two filter papers (Whatman No. 1) moistened with 4 mL of water or iminosugar solutions (500 μM), in 9 cm petri dishes (ten grains per dish), at 17 °C in the dark. Embryo-less half grains were incubated in 0.5 mL of the same solutions with or without 15 μM gibberellic acid (GA), individually in 20 mm^2^ wells of 25-well sterile plastic plates at 17 °C in the dark.

### Cloning, transient expression, immunoprecipitation and immunodetection of AXAH and endo-xylanase

Primers are described in Supplementary Table S1. Extraction of total RNA, first-strand cDNA synthesis and PCR amplification were as previously described^51^. The full length cDNAs of *HvAXAH1* and *HvAXAH2* (NCBI accessions JQ303075.1 and JQ303076.1 respectively^21^ were amplified with primers VA46 and VA47, and VA53 and VA54 respectively. The *HvXYN-1* coding sequence^19^ was commercially synthesised (Life Technologies; www.lifetechnologies.com/uk/) and amplified with primers VA36 and VA37. Constructs were cloned into pCR8/GW/TOPO vector, sequenced with primers VA48-VA51 (*HvAXAH1*), VA55 and VA56 (*HvAXAH2*), VA38 and VA39 (*HvXYN1*), and introduced by Gateway LR clonase II-mediated recombination (Life Technologies) in the following vectors: pGWB11 or pGWB14 or pGWB17 for C-terminal FLAG or 3xHA or 3x c-Myc epitope tags respectively^52^. Recombined binary plasmids were introduced into *Agrobacterium tumefaciens* strain GV3101. Bacteria were infiltrated into leaves of three-week-old *N. benthamiana* plants and extracts were prepared two days after infiltration^53^. Incubations with affinity matrices were performed at 4 °C with end-over-end mixing for 2 h with anti-FLAG^®^ M2, EZview™ Red anti-HA or EZview™ Red anti-c-Myc affinity matrices (Sigma-Aldrich). Bound proteins were collected and washed as described^53^. Following SDS-PAGE and transfer of proteins on nitrocellulose membranes, epitope-tagged chimeric proteins were detected with the following antibodies: anti-FLAG M2 (1/7500 dilution), anti-HA (clone HA-7; 1/7500 dilution), anti-c-Myc (clone 9E10; 1/12500 dilution) (Sigma-Aldrich) as appropriate, using the ECL chemiluminescence reagent (GE Healthcare).

### Protein chromatography

For ion exchange chromatography, the roots and coleoptile of 30–40 seedlings at ten dpi were removed and the remaining tissue (referred to as the endosperm), was rapidly frozen and stored at −80 °C until use. Tissue was ground under liquid nitrogen, homogenised in 100 mM MOPS/KOH (pH 7.2), 1 mM EDTA, 1 mM DTT, 10% (v/v) ethanediol, with protease inhibitors (plant cocktail, Sigma-Aldrich, UK], and filtered through two layers of muslin. Further procedures were at 4 °C. After clarification by centrifugation (20,000 *g* for 30 min), ammonium sulfate was added to the filtrate and protein precipitating between 20 and 80% saturation was collected by centrifugation as above. The pellet was resuspended in medium A [20 mM HEPES/KOH (pH 7.5), 5 mM CaCl_2_, 5% (v/v) ethanediol], desalted with Sephadex G25 (PD-10 columns, GE Healthcare) equilibrated with medium A, then applied to a MonoQ 5/50 GL anion exchange column (GE Healthcare) equilibrated with medium A, on an AKTA FPLC system (GE Healthcare). The column was washed with medium A at a flow rate of 1 ml min^−1^, then eluted with a 40-ml linear gradient of 0–1 M NaCl in medium A. Proteins in the initial wash (unbound MonoQ fraction) were desalted with Sephadex G25 equilibrated with medium B [25 mM Na acetate (pH 4.5), 5 mM CaCl_2_, 5% (v/v) ethanediol], then applied to a MonoS 5/50 GL cation exchange column equilibrated with medium B. The column was washed with medium B at a flow rate of 1 ml min^−1^, and eluted with a 40-ml linear gradient of 0–0.5 M NaCl in medium B.

For size exclusion chromatography, endosperm tissue prepared as above was homogenised in 50 mM Tris/HCl (pH 7.5), 150 mM NaCl, 1 mM DTT, with protease inhibitors (plant cocktail, Sigma-Aldrich). Following centrifugation and filtration through 0.22 μm filters, samples were applied to a Sephacryl S300HR 26/60 size exclusion column (GE Healthcare) equilibrated with 50 mM Tris/HCl (pH 7.5), 150 mM NaCl, at 1 ml min^−1^. Elution was with 1.5 column volumes (~480 ml) and 2-ml fractions were collected. The column was calibrated with protein standards in the range 29–700 kDa (Sigma-Aldrich) and the void volume determined with Blue Dextran (~2,000 kDa).

### Enzyme assays

Samples were desalted on Sephadex G25 (coarse) columns equilibrated with 50 mM sodium acetate (pH 5). Activity was measured in assays containing 50 mM sodium acetate (pH 4.5), as described for α-glucosidase^31^ but substituting the appropriate nitrophenyl glucosides (Megazyme, Ireland; Sigma-Aldrich) at 1.5 mM as follows: AXAH assay, 4-nitrophenyl α-l-arabinofuranoside (pNPA); endo-xylanase assay, 4-nitrophenyl xylotrioside (OpNPX3); β-d-xylosidase assay, 4-nitrophenyl β–d-xylanopyranoside (pNPX); β-glucosidase assay, 4-nitrophenyl β-d-glucopyranoside (β-pNPG); α-mannosidase assay, 4-nitrophenyl α-d-mannopyranoside (pNPman); β-galactosidase assay, 4-nitrophenyl β-d-galactopyranoside (pNPgal). Agl97 activity was assayed in assays containing 50 mM sodium acetate (pH 4.5), 5 mM CaCl_2_, 0.6 mM 4-nitrophenyl α-d-glucopyranoside (pNPG)^31^. Reactions were incubated at 37 °C for 1 h and terminated by the addition of an equal volume of 500 mM ammonium bicarbonate. Released p-nitrophenol was measured at 405 nm. α-Amylase and β-amylase activity were determined with the CERALPHA and BETAMYL kits respectively (Megazyme, Ireland). Limit dextrinase activity was assayed at 37 °C in assays containing 20 mM sodium acetate, pH 5.5, 5 mM CaCl_2_, 0.005% Triton X-100 (v/v) and 0.1 mg mL^−1^ pullulan^54^. Unless otherwise stated, inhibitors were included in reactions at 500 μM final concentration^31^.

### Microscopy

#### AX immunodetection

The roots and coleoptile of barley seedlings were removed and the remaining tissue immediately immersed in 2.5% (v/v) glutaraldehyde in 50 mM sodium cacodylate (pH 7.2), fixed, treated with osmium tetroxide and dehydrated^55^. Samples were infiltrated with LR White resin (London Resin Company Ltd., UK) over one week with daily changes of the resin, then infiltrated with fresh resin, polymerised at 60 °C for 16 h and sectioned on a Leica UC6 ultramicrotome (Leica Microsystems, Milton Keynes, UK). For the immunodetection of AX epitopes, 1 μm sections were blocked for 2 h with 5% (w/v) non-fat milk in phosphate buffered saline (PBS), incubated with LM11 antibody^35^ (PlantProbes, Leeds, UK), followed by incubation in the dark with an Alexa Fluor^®^ 633 conjugated secondary antibody (Life Technologies), both in 5% (w/v) milk powder in PBS for 1 h. Sections were extensively washed with fresh PBS and overlaid with AF1 glycerol-PBS antifadent solution (Citifluor, UK). For control sections the primary antibody was omitted. Detection of labelled epitopes by confocal microscopy was with a Leica SP5 scan head mounted on a Leica DM6000 upright microscope (excitation 632 nm, emission 647 nm).

#### Bright field and UV microscopy

Transverse sections of barley grains (1 μm) were stained with toluidine blue, counter-stained with Lugol’s iodine solution^56^ and viewed with a Leica DM6000 bright field microscope.

### Analytical procedures

#### Starch quantification

Extraction and measurement of starch was as previously described^31^. *AX oligosaccharide generation and fractionation*: The husk and embryo were removed from 30–40 dry grains or seedlings and the remaining tissue was pooled, ground under liquid nitrogen and freeze-dried. Samples (100 mg) were prepared as described^57^, then treated with 16 units of endo-xylanase M1 (EC 3.2.1.8) from *Trichoderma viride* (Megazyme)^57^,^58^. AX oligosaccharides were analysed by high-performance anion-exchange chromatography (HPAEC)^58^ on a Carbopac PA-1 column (2 × 250 mm; Dionex) at 25 °C and a flow rate of 1 ml min^−1^. Detection was with an ED40 electrochemical detector (Dionex) with an Au working electrode, and an Ag/AgCl reference electrode in pulsed amperometry mode. Xylose and xylan oligosaccharides (xylobiose to xylohexaose) were identified from the elution profile of standards (10 μM each; Megazyme). Peaks corresponding to AX oligosaccharides were identified through comparison with samples that had been digested with 2 units *Aspergillus niger* α-d-arabinofuranosidase (Megazyme) at 37 °C for 1 h.

## Additional Information

**How to cite this article**: Andriotis, V. M. E. *et al*. Cell wall degradation is required for normal starch mobilisation in barley endosperm. *Sci. Rep.* **6**, 33215; doi: 10.1038/srep33215 (2016).

## Supplementary Material

Supplementary Information
