# Supplementary material for: Cell wall degradation is required for normal starch mobilisation in barley endosperm
Source: Sci Rep. 2016 Sep 13;6:33215. doi: 10.1038/srep33215 (PMC5020691; doi:10.1038/srep33215)
Supplement: Supplementary Information [file srep33215-s1.pdf]

Supplementary Information

Cell wall degradation is required for normal starch mobilisation in barley endosperm

Vasilios M.E. Andriotis, Martin Rejzek, Elaine Barclay, Michael D. Rugen, Robert A. Field, Alison M. Smith

The following Supporting Information is available for this article:

Supplementary Fig. S1. Development of arabinoxylan-degrading activities in the endosperm.

Supplementary Fig. S2. Effects of LAB on cell wall-degrading activities in the endosperm.

Supplementary Fig. S3. Effects of LAB and xyloDNJ on seedling growth.

Supplementary Fig. S4. Identification of peaks diagnostic of AX content in cell wall fractions from endosperm.

Supplementary Fig. S5. Effect of LAB on arabinoxylan distribution in the endosperm at six dpi.

Supplementary Fig. S6. Size fractionation of AXAH and endoxylanase activities from barley endosperm.

Supplementary Fig. S7. Immunoprecipitation and activity of tagged versions of barley AXAH and endo-xylanase expressed transiently in *N. benthamiana* leaves.

Supplementary Fig. S8. Co-immunoprecipitation of tagged versions of barley AXAH and endoxylanase.

Supplementary Table S1. Primers used in this study.

Supplementary Fig. S1 Development of arabinoxylan-degrading activities in the endosperm. Grains were imbibed and grown in water. Extracts of endosperms were prepared from a pool of 10 endosperms at the indicated days post imbibition and used in assays with (**a**) pNPA, for AXAH activity, (**b**) OpNPX3, for endo-xylanase activity, (**c**) pNPX, for xylosidase activity. Values are means ± SE of measurements on three independent extracts. Error bars (SE) were smaller than the symbols. Data in (a) and (c) are from a different incubation from those in (b). Further information on the development of AXAH and endo-xylanase activities is provided in Figure S6.

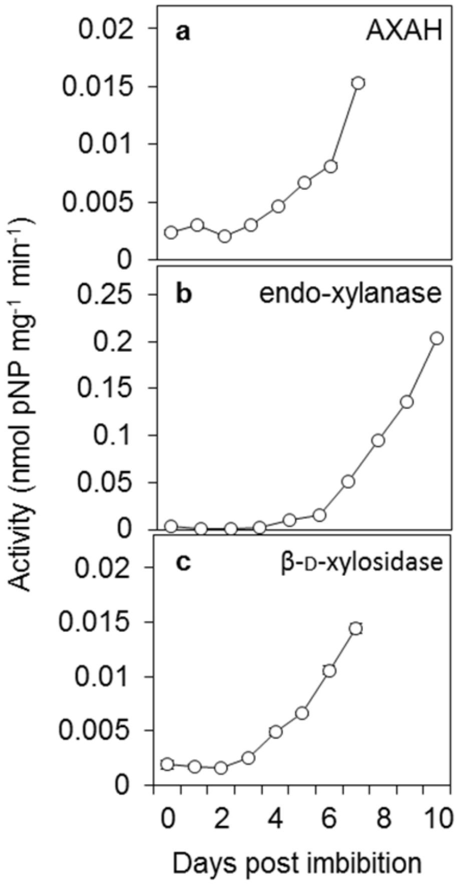

Supplementary Fig. S2 Effects of LAB on cell wall-degrading activities in the endosperm. Extracts of endosperms at 10 dpi were applied to a MonoQ anion exchange column (**a, c**) by FPLC. After washing, the column was eluted with a gradient of increasing NaCl concentration (dashed line) and fractions were collected. Proteins not binding to this column were applied to a MonoS cation exchange column (**b, d**) and eluted with a gradient of increasing NaCl concentration (dashed line). Enzyme activities were assayed either in the absence (circles) or in the presence of 500 μM LAB (triangles). (**a**) Elution profile of β-glucosidase activity (assayed with β-D-glucopyranoside) from a MonoQ column. A major peak of activity eluted at about 125 mM NaCl, and a second, poorly-resolved peak eluted at about 275-300 mM NaCl. LAB did not inhibit the β-glucosidase activity in either peak. (**b**) Elution profile from a MonoS column of β-glucosidase activity that did not bind to the MonoQ column in (**a**). This activity resolved in three peaks eluting at about 125 mM, 275-300 mM and 400 mM NaCl. LAB did not inhibit β-glucosidase activity in these peaks. (**c**) Elution profile from a MonoQ column of β-galactosidase (assayed with 4-nitrophenyl β-D-galactopyranoside). The activity eluted at about 100 mM NaCl, and was not inhibited by LAB. (**d**) Elution profile from a MonoS column of α-mannosidase activity (assayed with 4-nitrophenyl β-D-mannopyranoside) that did not bind to the MonoQ column in (**a**). The activity eluted at about 150-200 mM NaCl, and was not inhibited by LAB.

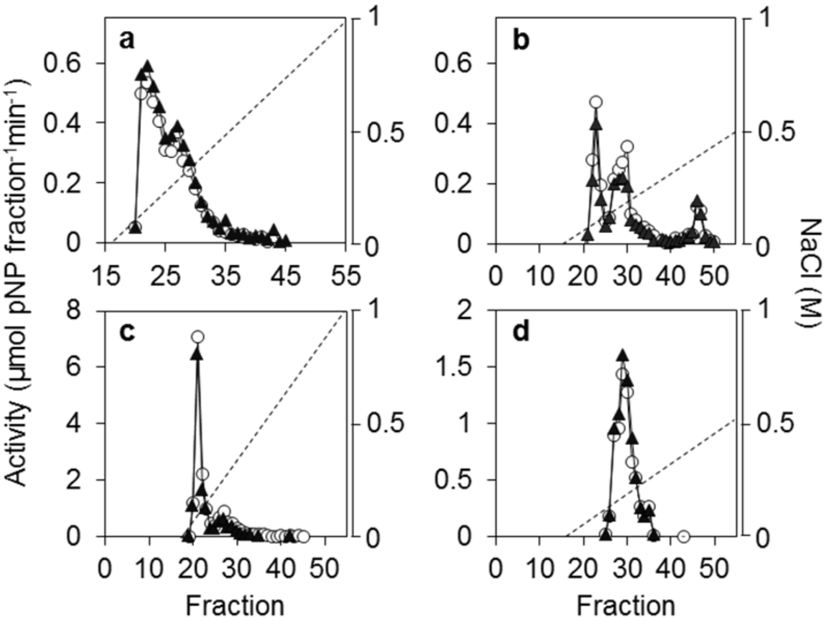

Supplementary Fig. S3 Effects of LAB and xyloDNJ on seedling growth. Appearance of seedlings of barley cv Tipple at ten dpi, grown in water alone or in the presence of either 200 μM or 500 μM LAB or xyloDNJ. Bar = 1 cm.

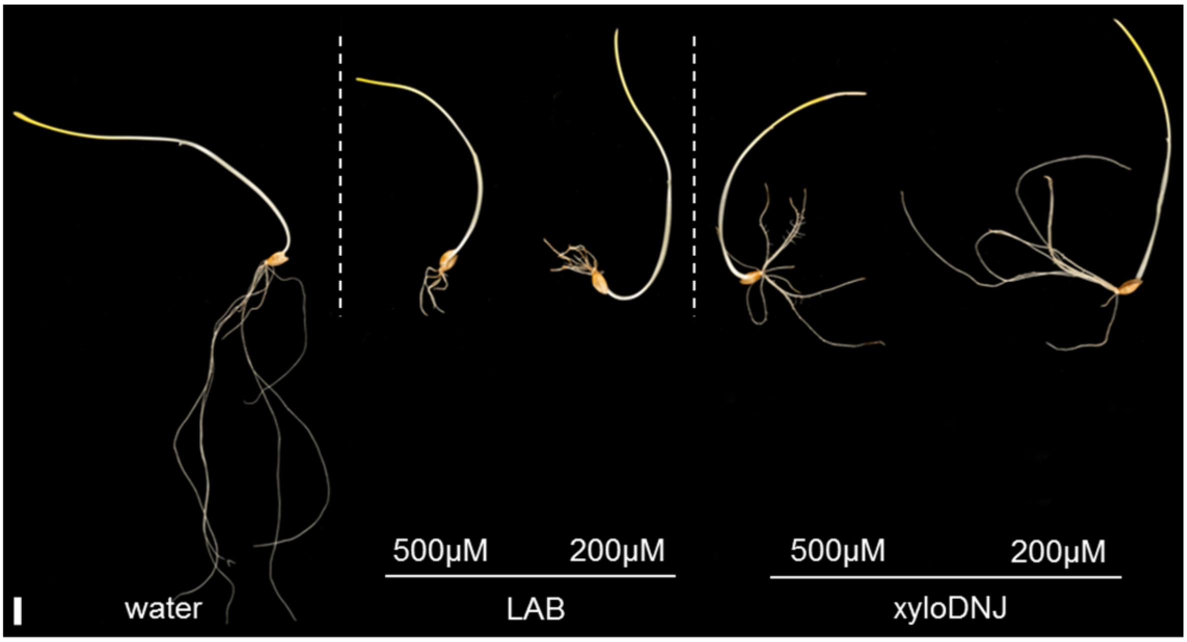

Supplementary Fig. S4 Identification of peaks diagnostic of AX content in cell wall fractions from endosperm. (**a**) The cell-wall fraction of the endosperm of dry grains was treated with endo-xylanase M1 from *Trichoderma viride*. Released AX oligosaccharides were then either digested with *Aspergillus niger* arabinofuranosidase [1 unit (U), middle panel or 2U, bottom panel], or not digested (0U) before HPEAC analysis. Based on dependence on arabinofuranosidase treatment, the peaks in the red box were identified as AX oligosaccharides. Numbers 1-4 at the top indicate peaks selected as diagnostic of AX content based on their good resolution, which allowed robust quantification. (**b**) Accumulation of arabinose (Ara) and xylose (Xyl) in cell-wall fraction of the endosperm of dry barley grains treated with endo-xylanase then digested with increasing amounts (0-2U) of arabinofuranosidase. The top three elution profiles correspond to the grey-shaded area in the elution profile in (**a**), and were compared with Ara and Xyl standards as shown. There was a very small detector response during runs without sample (blank, bottom panel). (**c**) Comparison of AX oligosaccharides in endosperm cell wall fractions from dry grains (top panel) and grains incubated in water for six days (bottom panel). The boxed area highlights major differences in the AX-containing peaks identified in (**a**). Numbers 1-4 on the top indicate peaks selected as diagnostic of AX content [see (**a**)].

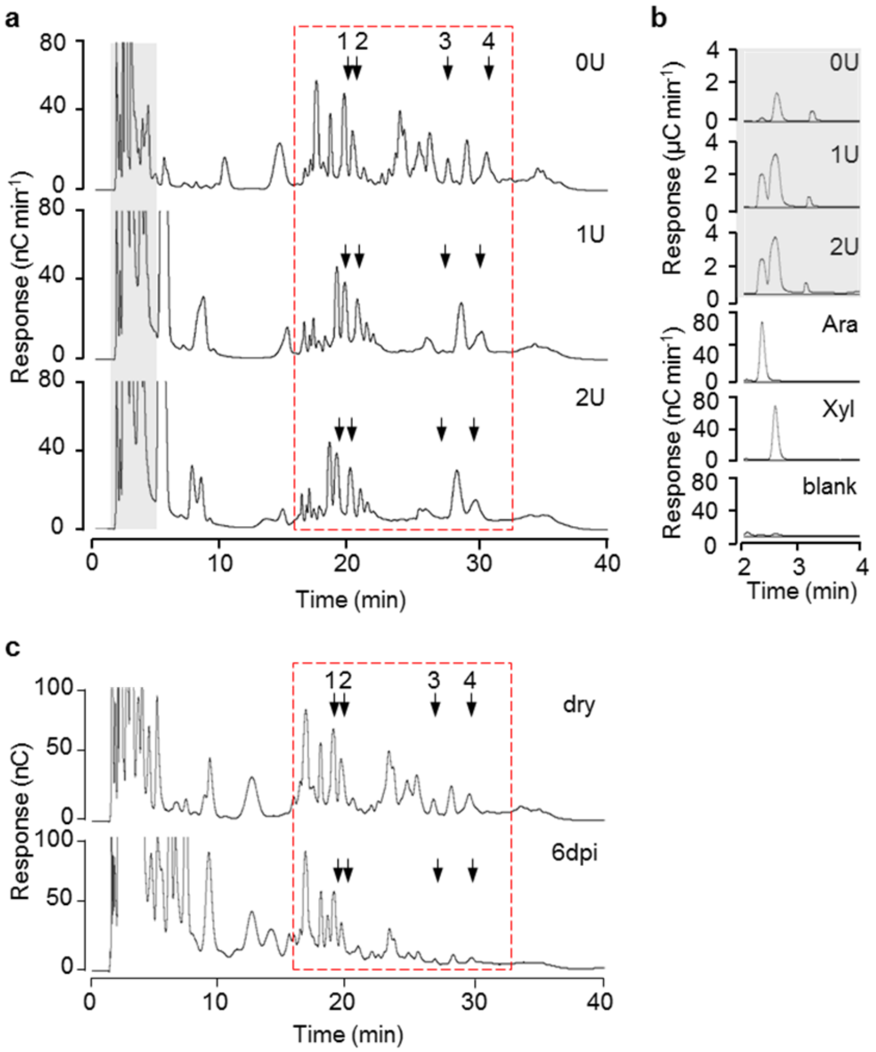
a
b
c
Response (nC min-1)
Time (min)
0U
1U
2U
1 2
3
4
Response (µC min-1)
Ara
Xyl
blank
Response (nC)
dry
6dpi
12

Supplementary Fig. S5 Effect of LAB on arabinoxylan distribution in the endosperm at six dpi. Transverse sections of endosperm of grains imbibed and grown in water (**a**), or LAB (500 µM; **b**) were incubated with monoclonal antisera against AX (LM11) then with an Alexa Fluor® 633 conjugated secondary antibody, and subjected to confocal microscopy to visualise AX epitopes. Sections were prepared from approximately the same position of the grain, and are from different grains from those shown in Fig. 6. Arrowheads point to fluorescence (red) from AX epitopes in endosperm cell walls, arrows point to the aleurone layer, and the dashed line indicates the crease of the grain. Scale bars = 500 µm.

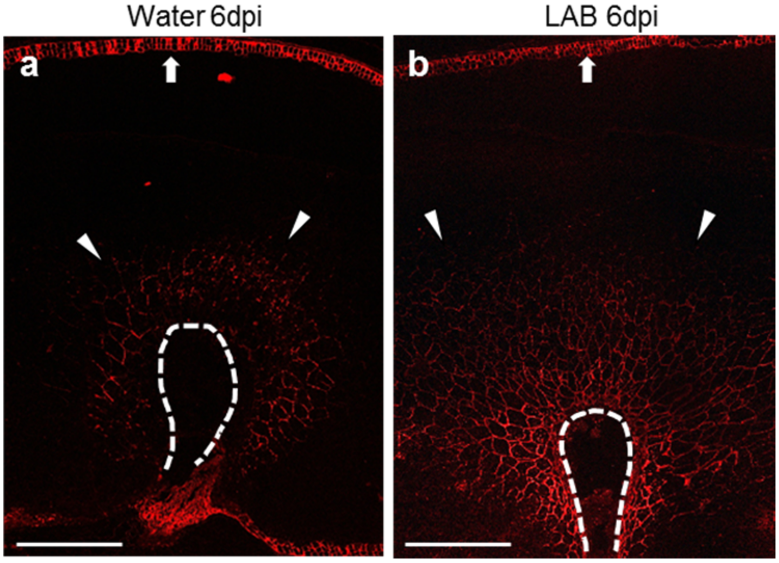

Supplementary Fig. S6 Size fractionation of AXAH and endo-xylanase activities from endosperm extracts. Extracts from the endosperm of barley seedlings at 4, 6, 8, and 10 dpi were applied to a Sephacryl S200 column by FPLC. Fractions (2 ml) were collected and assayed for AXAH and endo-xylanase activity. Numbers on the top of each panel indicate the elution positions of proteins of known molecular mass (in kDa). Vo is the void volume of the column, estimated by the elution volume of Blue Dextran 2000. (**a**) AXAH activity (assayed with pNPA). (**b**) Endo-xylanase activity (assayed with OpNPX3). I-III indicate the three peaks of activity. The inset in the top panel is a magnification of the area highlighted (red dashed-line box) in the same panel.

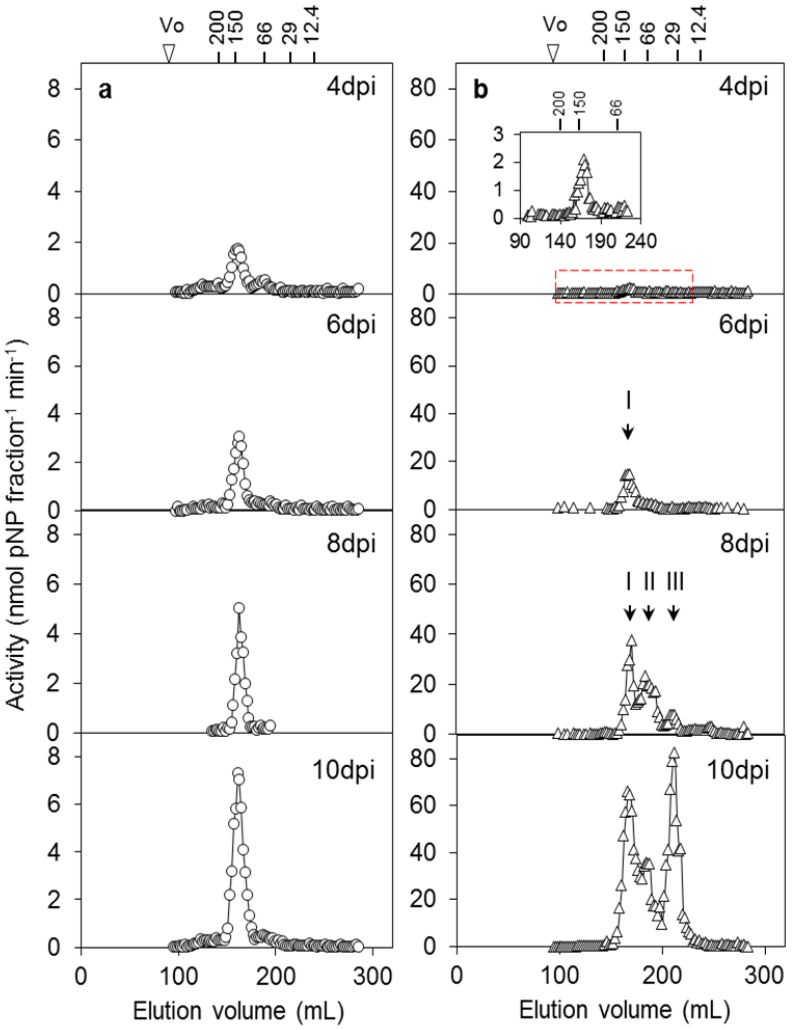

Supplementary Fig. S7 Immunoprecipitation and activity of tagged versions of barley AXAH and endo-xylanase expressed transiently in *N. benthamiana* leaves. (**a-c**) Accumulation of (**a**) AXAH1-FLAG and AXAH1-3xHA, (**b**) AXAH2-3xHA and AXAH2-3xcMyc, and (**c**) pXYN1-FLAG and pXYN-3xcMyc in *N. benthamiana* leaves. Extracts were prepared either from uninfiltrated areas of leaves or from areas transiently expressing epitope-tagged proteins. Samples were subjected to SDS-PAGE followed by immunoblot analysis with antibodies (Ab) specific for the cognate epitope tag (shown at the bottom of each panel). An equal amount of protein was loaded for each sample. The results verify the specific accumulation of tagged proteins of the expected size (closed arrowheads) in infiltrated areas. The open arrowhead in (**a**) indicates a non-specific band present in extracts of both infiltrated and uninfiltrated areas. This band was not precipitated by the affinity matrix for the FLAG epitope. The open arrowhead in (**c**) indicates the position of a cross-reacting band occasionally detected with both α-FLAG and α-cMyc antibodies in samples from infiltrated areas only. Numbers on the left indicate positions of molecular mass markers (mass in kDa). (**d-f**) Specific and quantitative immunoprecipitation of (**d**) AXAH1-FLAG (top) and AXAH1-3xHA (bottom), (**e**) AXAH2-3xHA (top) and AXAH2-3cMyc (bottom), and (**f**) XYN-1-FLAG (top) and XYN-1-3xcMyc (bottom) proteins transiently expressed in *N. benthamiana* leaves. Extracts were prepared as in (**a-c**) and used in immunoprecipitation (IP) assays with specific affinity matrices for each epitope tag. Proteins were detected with antibodies (Ab) specific for each epitope tag, as indicated at the bottom of each panel. No signal was detected with extracts from uninfiltrated leaf areas. Numbers on the left of each panel indicate positions of molecular mass markers (mass in kDa). In: input; U: soluble fraction remaining after IP. Closed arrowheads indicate the position of proteins of expected size. Asterisk: position of the IgG heavy chain occasionally detected by the antibodies. (**g-i**) Activity of chimeric proteins transiently expressed in *N. benthamiana* leaves. Extracts were prepared either from mock-infiltrated leaf areas (grey bars) or a pool of 4-5 leaves transiently expressing (**g**) AXAH1-FLAG or AXAH1-3xHA, (**h**) AXAH2-3xHA or AXAH2-3cMyc, (**i**) XYN-1-FLAG or XYN-1-3xcMyc proteins. AXAH activity was assayed with pNPA, and endo-xylanase activity with OpNPX3. Values are means ± SE of three independent measurements on a single extract.

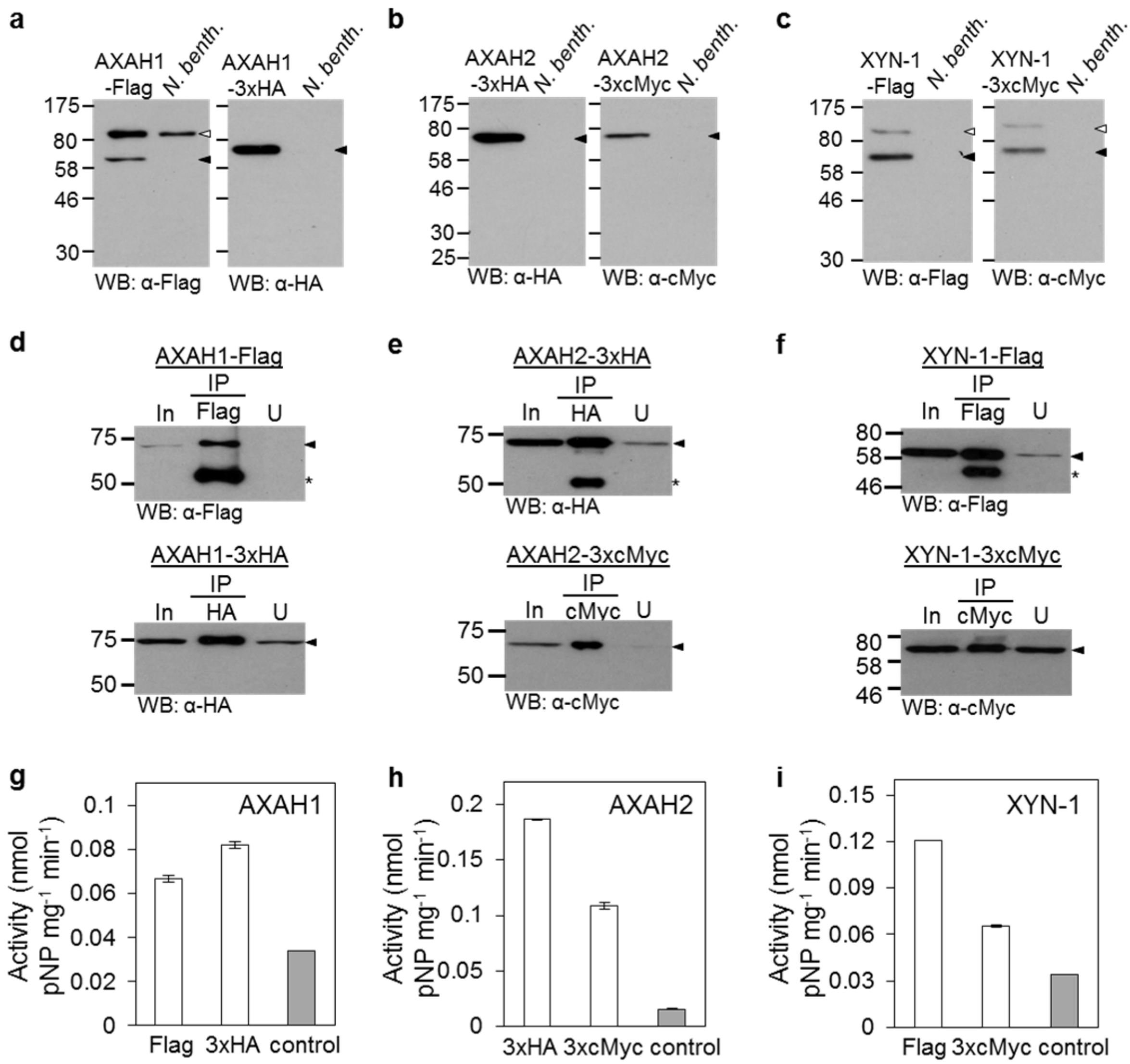

a
AXAH1 -Flag
N. benth.
AXAH1 -3xHA
N. benth.
175
80
58
46
30
WB: α-Flag
WB: α-HA
b
AXAH2 -3xHA
N. benth.
AXAH2 -3xcMyc
N. benth.
175
80
58
46
30
25
WB: α-HA
WB: α-cMyc
c
XYN-1 -Flag
N. benth.
XYN-1 -3xcMyc
N. benth.
175
80
58
46
30
WB: α-Flag
WB: α-cMyc
d
AXAH1-Flag
IP
In
Flag
U
75
50
WB: α-Flag
AXAH1-3xHA
IP
In
HA
U
75
50
WB: α-HA
e
AXAH2-3xHA
IP
In
HA
U
75
50
WB: α-HA
AXAH2-3xcMyc
IP
In
cMyc
U
75
50
WB: α-cMyc
f
XYN-1-Flag
IP
In
Flag
U
80
58
46
WB: α-Flag
XYN-1-3xcMyc
IP
In
cMyc
U
80
58
46
WB: α-cMyc
g
AXAH1
Activity (nmol pNP mg-1 min-1)
0.1
0.08
0.06
0.04
0.02
0
Flag
3xHA
control
h
AXAH2
Activity (nmol pNP mg-1 min-1)
0.2
0.15
0.1
0.05
0
3xHA
3xcMyc
control
i
XYN-1
Activity (nmol pNP mg-1 min-1)
0.15
0.12
0.09
0.06
0.03
0
Flag
3xcMyc
control

Supplementary Fig. S8 Co-immunoprecipitation of tagged versions of barley AXAH and endo-xylanase. Forms of AXAH2 and XYN-1 with epitope tags at the C-terminus were transiently expressed in *N. benthamiana* leaves. + above a lane indicates that the protein indicated at the left was expressed in the leaf from which extracts were made. Input samples are extracts prior to immunoprecipitation; IP: α-FLAG and IP: α-HA are proteins precipitated with the FLAG and HA antisera respectively. Ab: α-FLAG and Ab: α-HA indicate the antiserum used for immunodetection of proteins following SDS-PAGE. Numbers on the left are positions of molecular mass markers (in kDa). Numbers below each panel indicate lane numbers. Samples were subjected to electrophoresis on the same gel and to immunoblot analysis at the same time. Asterisk: position of the IgG heavy chain occasionally detected by the antisera. **(a)** AXAH2 can homodimerize. AXAH2-3xHA and/or AXAH2-3cMyc were transiently expressed in *N. benthamiana* leaves. **(b)** XYN-1 can homodimerize. XYN-1-FLAG and/or XYN-1-3xcMyc were transiently expressed in *N. benthamiana* leaves. Open arrowhead: position of a cross-reacting band present in extracts from leaves expressing XYN-1-FLAG (panel on the left; lanes 1,3). **(c)** AXAH2 does not interact with XYN-1. AXAH2-3xHA and/or XYN-1-FLAG were transiently expressed in *N. benthamiana* leaves.

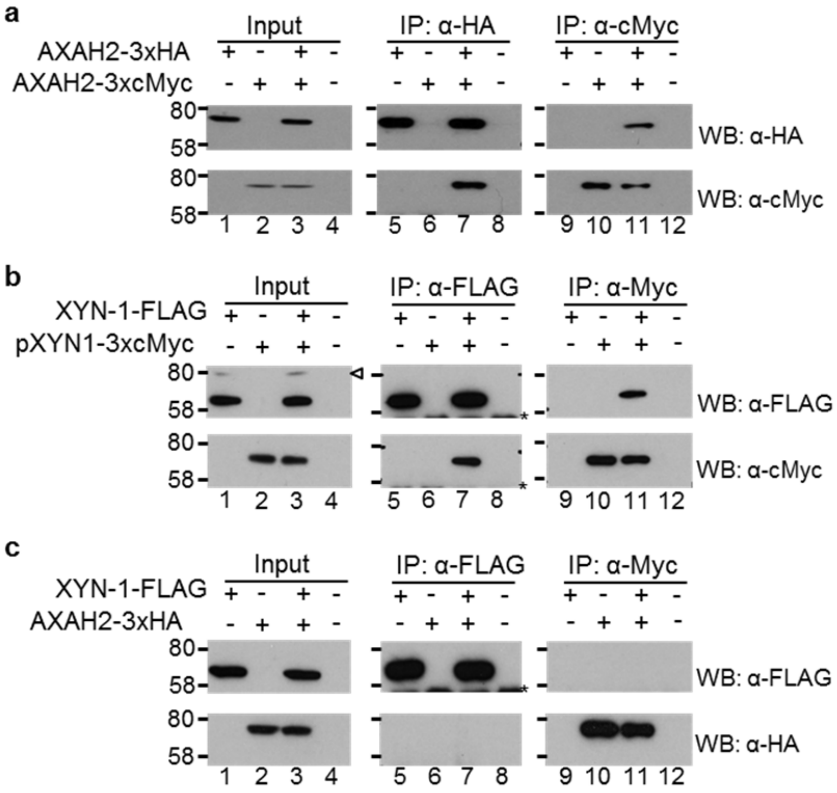

Supplementary Table 1. Primers used in this study.

Primer	Sequence[a]	Complement[b]	Description
VA36	ATGGCTTCTACTACCCAGGATGTG	FW	*HvXYN1* cloning
VA37	AGCATCAGCCAAATCCACAACCACAGG	RV	*HvXYN1* cloning
VA38	TTCTGGTCCGTTGATGGTGA	FW	*HvXYN1* internal primer, sequencing
VA39	CGTGCAGCATCTCGTTGTTA	RV	*HvXYN1* internal primer, sequencing
VA46	ATGGGTTCCAAAGAAATGCCCC	FW	*HvAXAH1* cloning
VA47	CACGTCCGCTACAAGCCTGGAC	RV	*HvAXAH1* cloning
VA48	TGCTTTGTTGAAGGCAGTTG	FW	*HvAXAH1* internal primer, sequencing
VA49	CCGGTTCCAGTGCACTATTT	FW	*HvAXAH1* internal primer, sequencing
VA50	GTGCATAGCTTGCCATCTGA	RV	*HvAXAH1* internal primer, sequencing
VA51	TGGCTGATTCCATTGTTGAA	RV	*HvAXAH1* internal primer, sequencing
VA53	ATGGGTTCCAAACAAGTGTTCGTT	FW	*HvAXAH2* cloning
VA54	CATCTCTGCGACAAGCTCGGA	RV	*HvAXAH2* cloning
VA55	GGGGAGTGCAAACTCAACAT	FW	*HvAXAH2* internal primer, sequencing
VA56	GCAGGATGGTCAAGTGGTTT	RV	*HvAXAH2* internal primer, sequencing

[a] Oligonucleotide sequences are shown in the 5’→3’ direction.

[b] FW, forward; RV, reverse
